# Supplementary material for: High performance floating self-excited sliding triboelectric nanogenerator for micro mechanical energy harvesting
Source: Nat Commun. 2021 Aug 3;12:4689. doi: 10.1038/s41467-021-25047-y (PMC8333367; doi:10.1038/s41467-021-25047-y)
Supplement: Supplementary file 1 — Supplementary Information [file 41467_2021_25047_MOESM1_ESM.pdf]

Supplementary Information for

High performance floating self-excited sliding triboelectric nanogenerator for micro mechanical energy harvesting

Long et al.

Supplementary Fig. 1: The photograph of the VMC.

Supplementary Fig. 2: Initial charge without charge self-excitation.

Supplementary Fig. 3: Schematic diagram of the slider with two pairs of excitation electrodes reaching a stable state.

Supplementary Fig. 4: The output performance of different pairs of electrodes for slider with charge self-excitation.

Supplementary Fig. 5: Initial charge of different pairs of electrodes for TENG without charge self-excitation.

Supplementary Fig. 6: Charge output increment of the electrode with different electrode length-width with charge self-excitation under air gap is 1 mm.

Supplementary Fig. 7: Initial charge of the electrode with different electrode areas and air gaps without charge self-excitation.

Supplementary Fig. 8: Dynamic output charge of one electrode FSS-TENG.

Supplementary Fig. 9: Dynamic output charge of FSS-TENG with different materials (FEP, PTFE and Kapton) in 0.35 mm air gap.

Supplementary Fig. 10: The excitation voltage of FSS-TENG under different capacitors.

Supplementary Fig. 11: The surface charge density of FSS-TENG under different temperature **a** and humidity **b**.

Supplementary Fig. 12. The surface charge density of FSS-TENG under the simulated solar light.

Supplementary Fig. 13: Structure and performance of floating TENG (F-TENG).

Supplementary Fig. 14: Voltage curves of capacitors charging by F-TENG at 300 rpm.

Supplementary Fig. 15: Stability test of F-TENG.

Supplementary Fig. 16: The output charge curves for the first hour and the last hour.

Supplementary Fig. 17: SEM images of unused film.

Supplementary Fig. 18: Device abrasion photographs of the stator of S-TENG after 100 thousand times cycles.

Supplementary Table 1: The comparison of charge density with the reported works.

Supplementary Table 2: The rotational speed of the rotator at different wind speeds.

Supplementary Movie 1: Demonstration of the dynamic process of charge self-

excitation driven by wind.

Supplementary Movie 2: Demonstration of 912 LEDs lit by the FSS-TENG at 5 m s^{-1} wind speed.

Supplementary Movie 3: Demonstration of the simulated road warning lights driven by the FSS-TENG at 5 m s^{-1} wind speed.

Supplementary Movie 4: Demonstration of two temperature hygrometers in parallel driven by the FSS-TENG at 3 m s^{-1} wind speed.

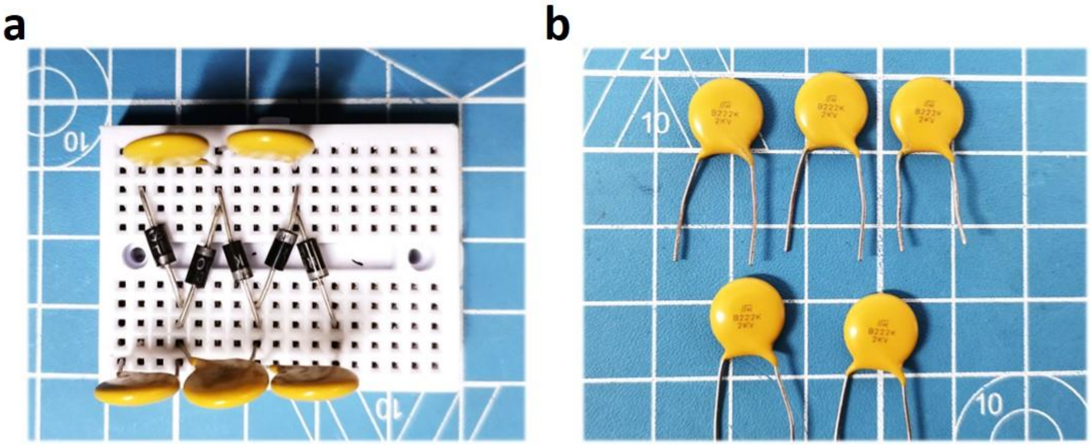

Supplementary Fig. 1 The photograph of the VMC. a The connecting circuit of VMC. **b** Ceramic capacitor.

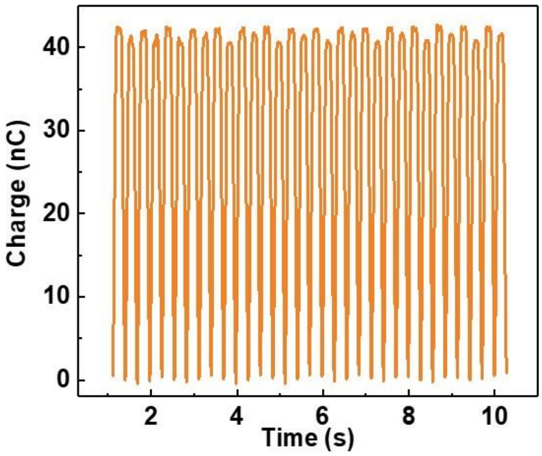

Supplementary Fig. 2 Initial charge without charge self-excitation.

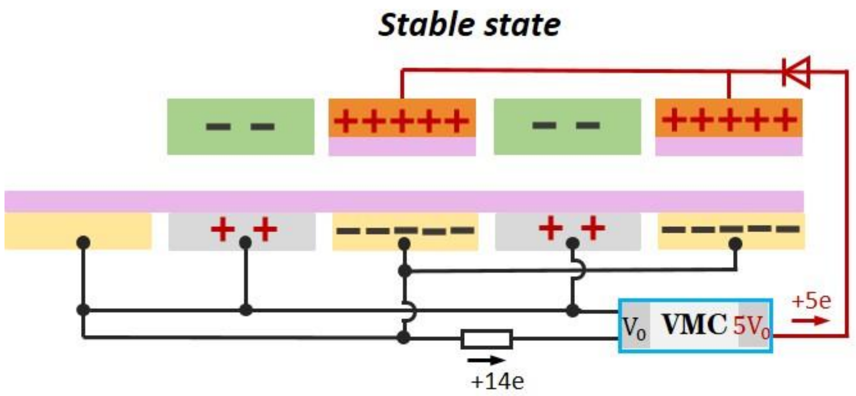

Supplementary Fig. 3 The schematic diagram of the slider with two pairs of excitation electrodes reaching a stable state.

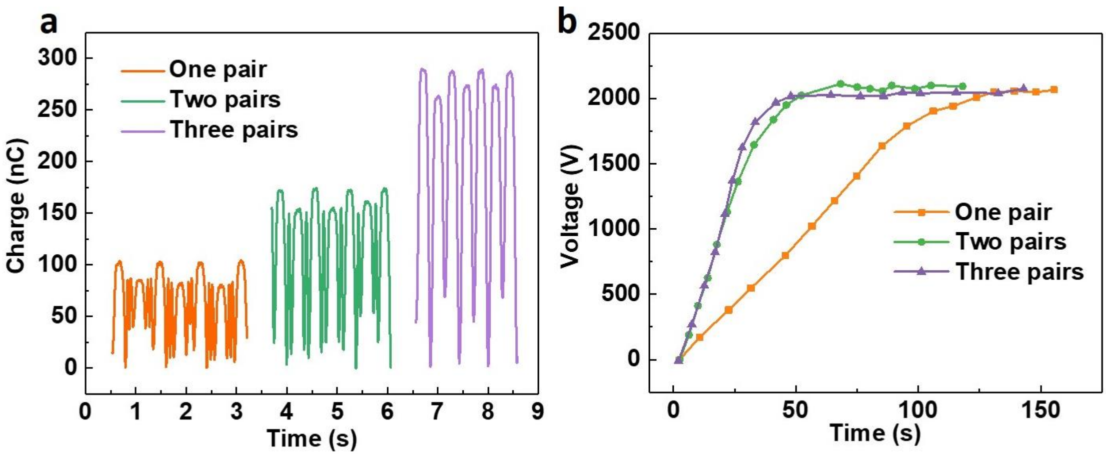

Supplementary Fig. 4 The output performance of different pairs of sliders (a PTFE and an electrode in one pair) with charge self-excitation. a The transferred charges and **(b)** excitation voltage.

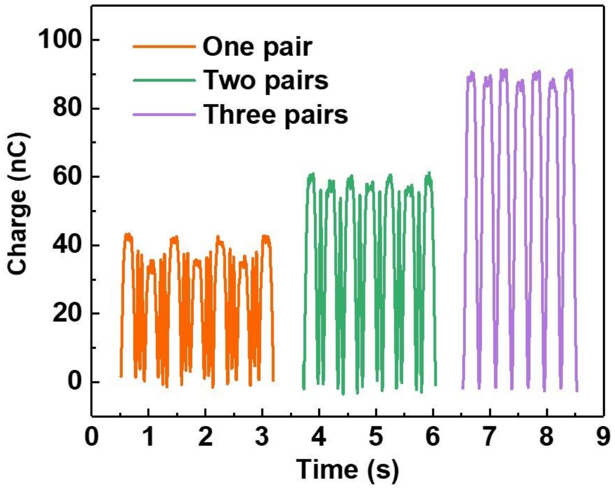

Supplementary Fig. 5 Initial charge of different pairs of sliders for the TENG without charge self-excitation.

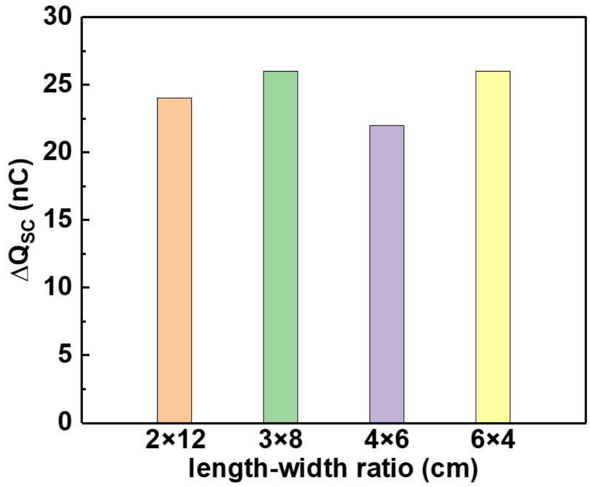

Supplementary Fig. 6 Charge output increment of the electrode with different electrode length-width ratio with charge self-excitation under air gap is 1 mm. The surface of the stator electrode is coated with Kapton film, and the slider electrode is none.

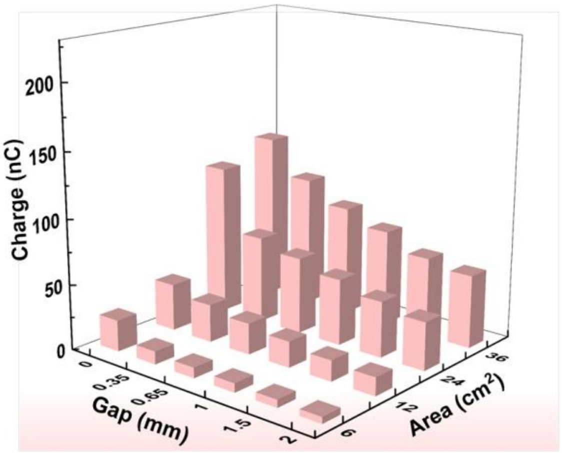

Supplementary Fig. 7 Initial charge of the electrode with different electrode areas and air gaps without charge self-excitation. The surface of the stator electrode is coated with Kapton film, and no film is for the slider electrode.

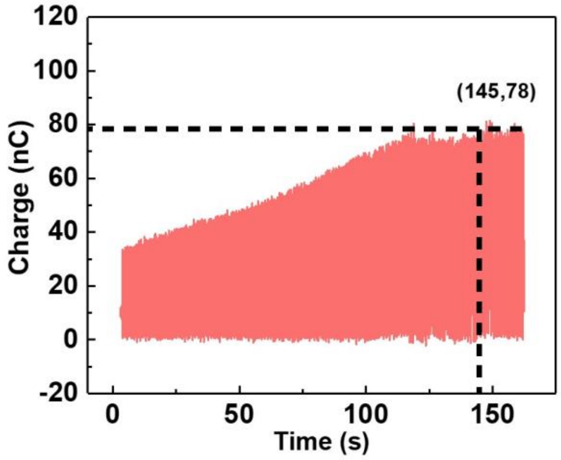

Supplementary Fig. 8 Dynamic output charge of one electrode FSS-TENG. No dielectric film is attached to the Cu electrode of the slider, and a 25μm Kapton film is attached to the Al electrode on the stator.

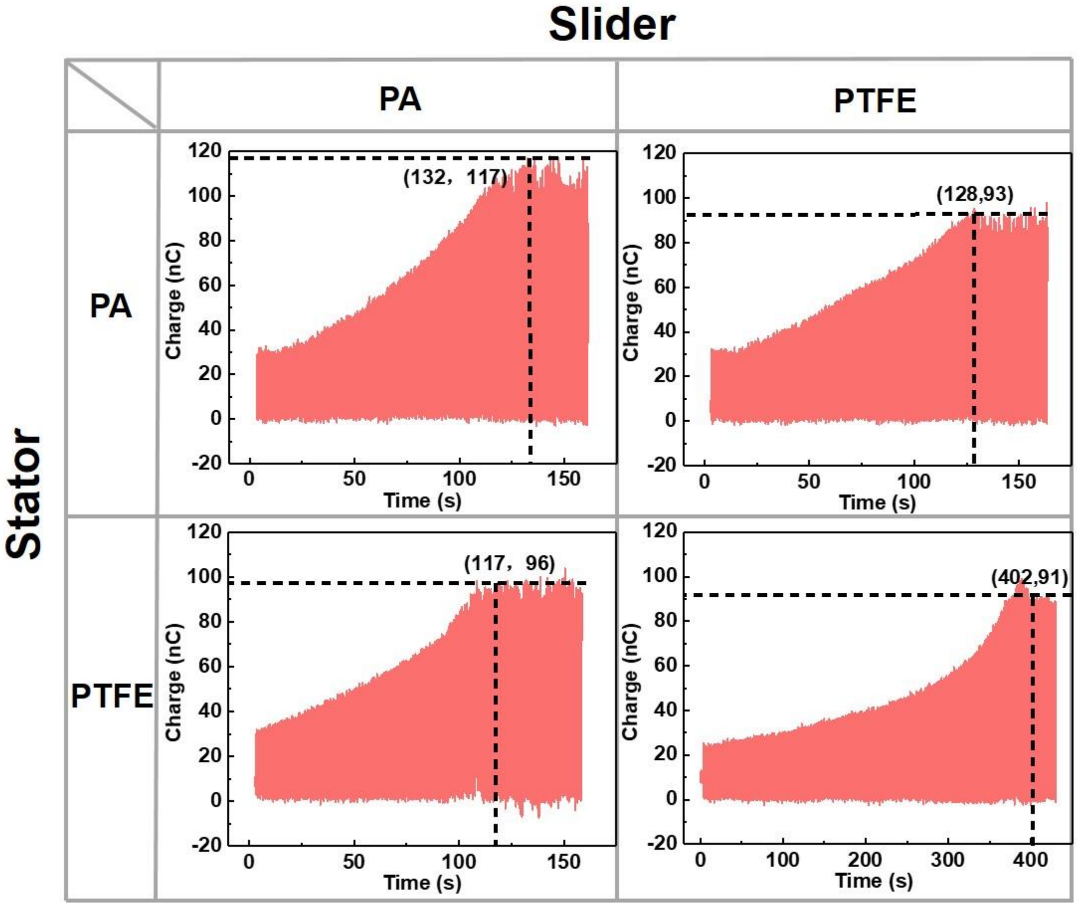

Supplementary Fig. 9 Dynamic output charge of FSS-TENG with different materials (FEP/ PTFE / Kapton) in 0.35 mm air gap.

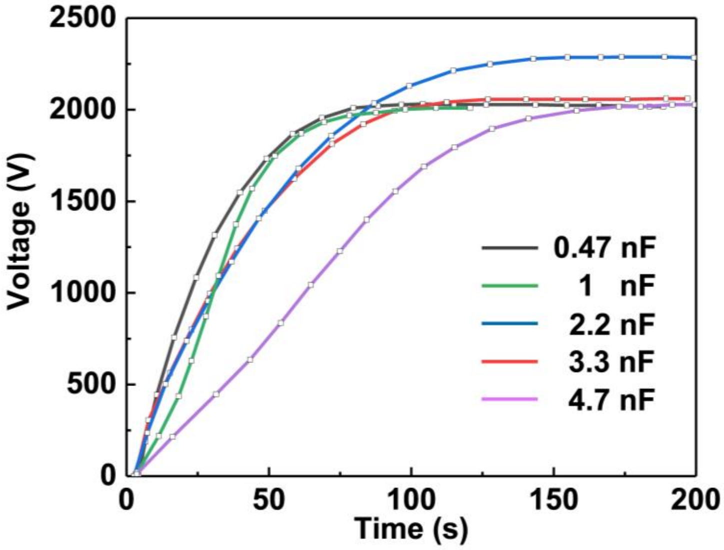

Supplementary Fig. 10 The excitation voltage of FSS-TENG under different capacitors.

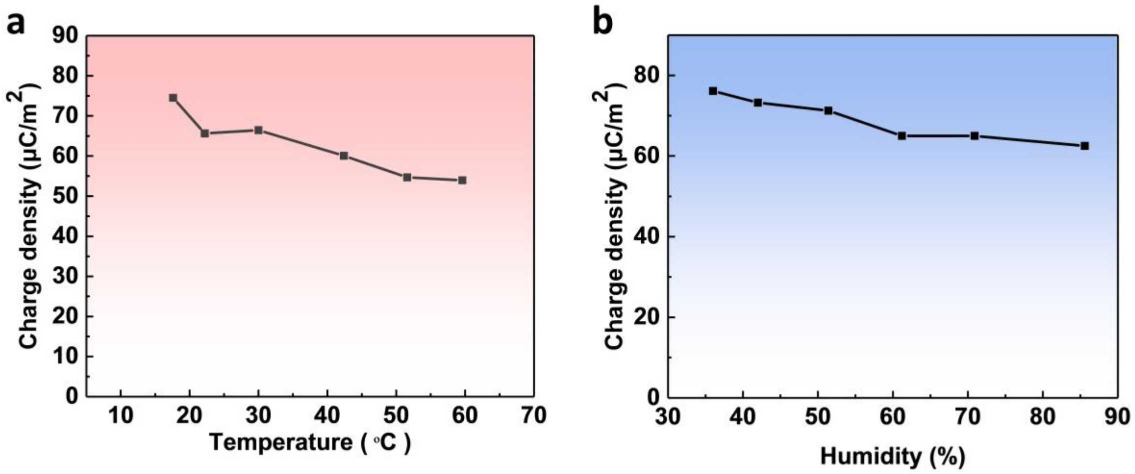

Supplementary Fig. 11 The surface charge density of FSS-TENG under different temperature (a) and humidity (b). a The test humidity is controlled at around 40%. **b** The test temperature is controlled at around 20 °C.

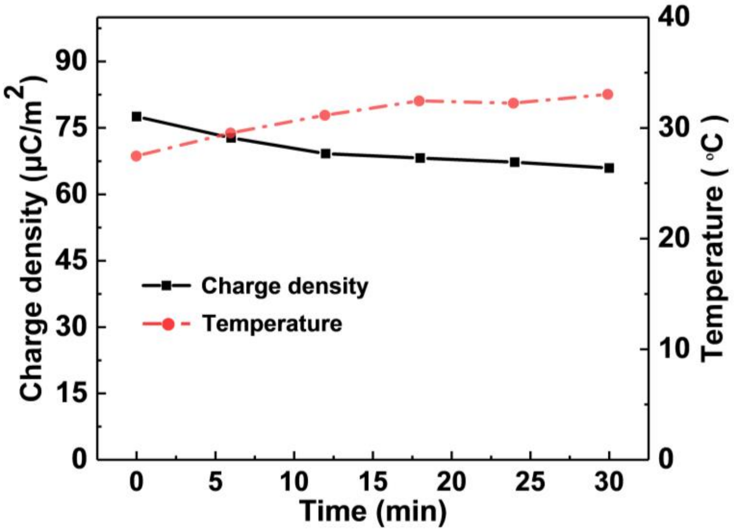

Supplementary Fig. 12 The surface charge density of FSS-TENG under the simulated solar light. The standard light intensity is 100 mW/cm^2. The test humidity is controlled at around 50%.

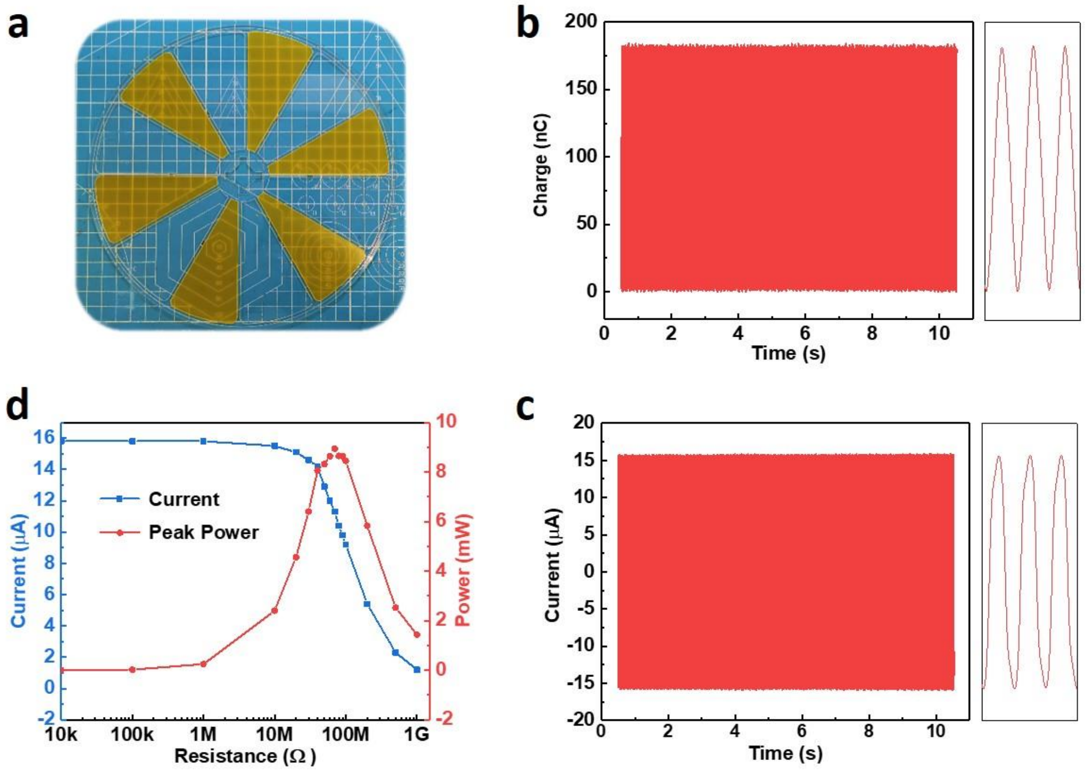

Supplementary Fig. 13 Structure and performance of floating TENG (F-TENG). **a** Device photographs of the rotator. The stator is the same as FSS-TENG. **b** Transferred charge and (**c**) current curves of the F-TENG at 300 rpm, respectively. The right side of the figure is the enlarged output curve. **d** Matching impendence and output power of F-TENG at 300 rpm.

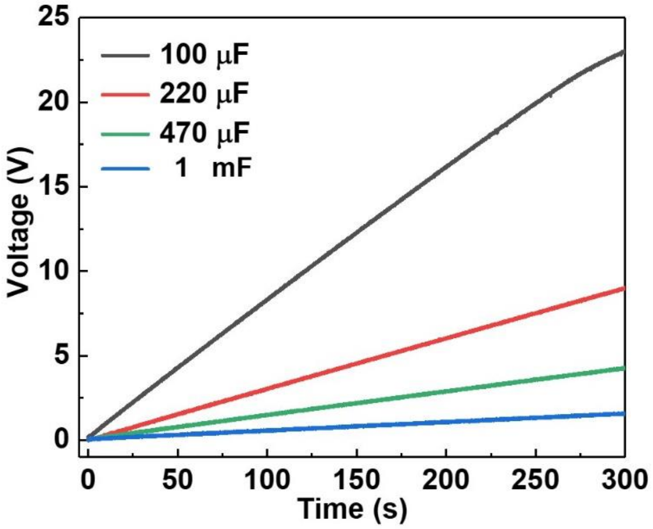

Supplementary Fig. 14 Voltage curves of capacitors charging by F-TENG at 300 rpm.

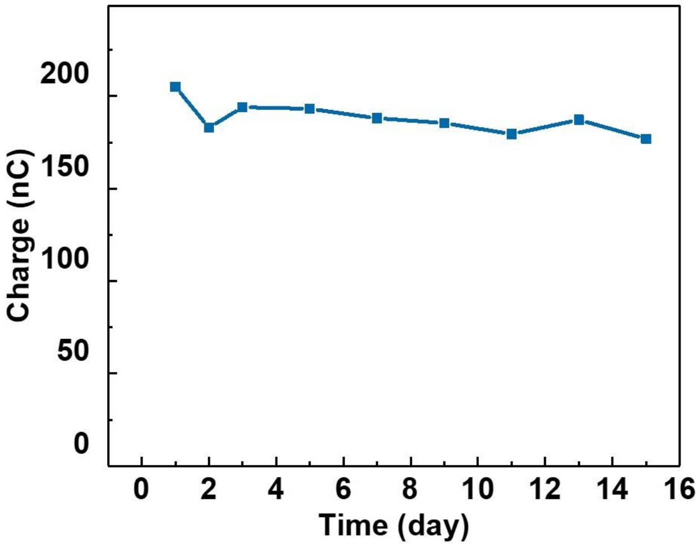

Supplementary Fig. 15 Stability test of F-TENG. We tested the induced output charge of F-TENG intermittently of 15 days. And the ambient humidity is controlled at 40% to 50%.

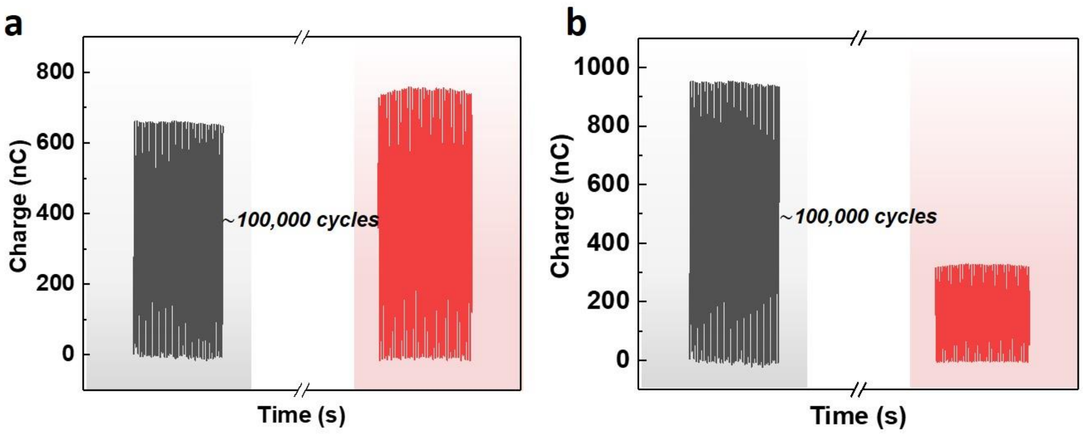

Supplementary Fig. 16 The output charge curves for the first hour and the last hour. a The stability of FSS-TENG. **b** The stability of S-TENG.

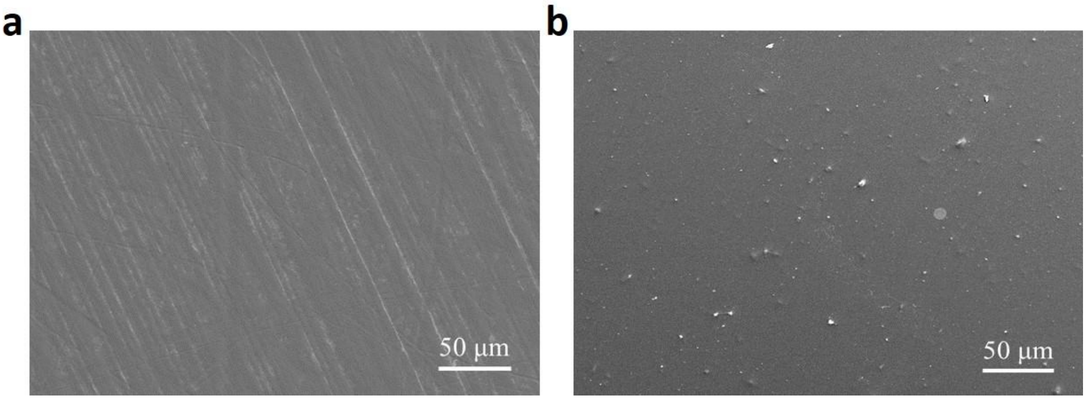

Supplementary Fig. 17 SEM images of unused film. a PTFE and **b** PA.

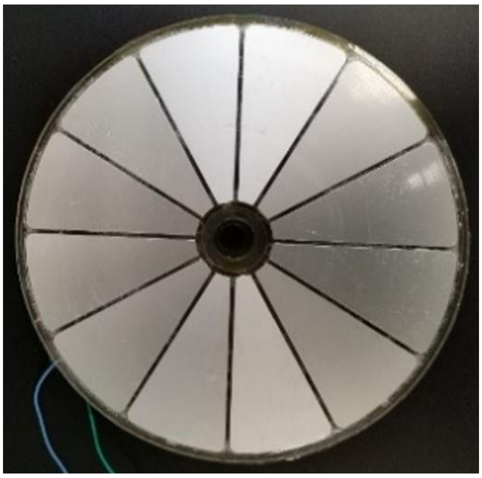

Supplementary Fig. 18 Device abrasion photographs of stator of S-TENG after 100 thousand times cycles.

Supplementary Table 1. The comparison of charge density with the reported works.

Boosting output charge of non-contact TENG				
**Charge supplementary**	**Gap(mm)**	Q_{SC} **(nC)**	$\sigma_{SC}\ (\mu Cm^{-2})$	**Related work**
no	a certain	46	7.7	Appl. Phys. Rev. 7, 021401 (2020)
no	5	no	25	Adv. Mater.2014, 26, 2818–2824
no	0.5	no	12.2	Adv. Mater. 2014, 26, 6599–6607
no	1	6.9	1.6	Nano Res. 13,1903–1907(2020)
no	0.5	no	20	ACS Appl. Mater. Interfaces 2014,6, 3031–3038
PTFE stripes	1.5	256	16.3	Adv. Energy Mater. 2020, 10, 2000064
Rabbit hair brushes	< 3	135	> 20.3	Nano Energy 81 (2021) 105625
PTFE stripes	1	182	18.9	Nano Energy 64 (2019) 103908
Charge Self-excitation	0.35	1000	71.5	This work

Supplementary Table 2. The rotational speed of the rotator at different wind speeds.

**Wind speed ($m\ s^{-1}$)**	3	4	5	6	6.5	7
**rotational speed (rpm)**	19.74	71.43	99.24	117.18	131.78	139.34

At different wind speeds, the rotator speed is obtained by calculating the cycle number of measured transfer charge per second.
