# Supplementary material for: High performance floating self-excited sliding triboelectric nanogenerator for micro mechanical energy harvesting
Source: Nat Commun. 2021 Aug 3;12:4689. doi: 10.1038/s41467-021-25047-y (PMC8333367; doi:10.1038/s41467-021-25047-y)
Supplement: Supplementary file 3 — Description of Additional Supplementary Files [file 41467_2021_25047_MOESM3_ESM.docx]

**Description of Additional Supplementary Files**

**Supplementary Movie 1:** Demonstration of the dynamic process of charge self-excitation driven by wind.

**Supplementary Movie 2:** Demonstration of 912 LEDs lit by the FSS-TENG at 5 m s^-1^ wind speed.

**Supplementary Movie 3:** Demonstration of the simulated road warning lights driven by the FSS-TENG at 5 m s^-1^ wind speed.

**Supplementary Movie 4:** Demonstration of two temperature hygrometers in parallel driven by the FSS-TENG at 3 m s^-1^ wind speed.
